# Safety of a condom uterine balloon tamponade (ESM-UBT) device for uncontrolled primary postpartum hemorrhage among facilities in Kenya and Sierra Leone

**DOI:** 10.1186/s12884-018-1808-z

**Published:** 2018-05-15

**Authors:** Aparna Ramanathan, Melody J. Eckardt, Brett D. Nelson, Moytrayee Guha, Monica Oguttu, Zaid Altawil, Thomas Burke

**Affiliations:** 1 Division of Global Health and Human Rights, Department of Emergency Medicine, Massachusetts General Hospital, 125 Nashua St, Suite 910, Boston, MA 02114 USA; 2 Harvard T.H. Chan School of Public Health, Boston, MA USA; 3 Harvard Medical School, Boston, MA USA; 4 Kisumu Medical and Education Trust, Kisumu, Kenya

**Keywords:** Uterine balloon tamponade, Postpartum hemorrhage, Maternal mortality, Maternal health, Developing countries

## Abstract

**Background:**

Postpartum hemorrhage is the leading cause of maternal mortality in low- and middle-income countries. While evidence on uterine balloon tamponade efficacy for severe hemorrhage is encouraging, little is known about safety of this intervention. The objective of this study was to evaluate the safety of an ultra-low-cost uterine balloon tamponade package (named ESM-UBT) for facility-based management of uncontrolled postpartum hemorrhage (PPH) in Kenya and Sierra Leone.

**Methods:**

Data were collected on complications/adverse events in all women who had an ESM-UBT device placed among 92 facilities in Sierra Leone and Kenya, between September 2012 and December 2015, as part of a multi-country study. Three expert maternal health investigator physicians analyzed each complication/adverse event and developed consensus on whether there was a potential causal relationship associated with use of the ESM-UBT device. Adverse events/complications specifically investigated included death, hysterectomy, uterine rupture, perineal or cervical injury, serious or minor infection, and latex allergy/anaphylaxis.

**Results:**

Of the 201 women treated with an ESM-UBT device in Kenya and Sierra Leone, 189 (94.0%) survived. Six-week or longer follow-up was recorded in 156 of the 189 (82.5%). A causal relationship between use of an ESM-UBT device and one death, three perineal injuries and one case of mild endometritis could not be completely excluded. Three experts found a potential association between these injuries and an ESM-UBT device highly unlikely.

**Conclusion:**

The ESM-UBT device appears safe for use in women with uncontrolled PPH.

**Trial registration:**

Trial registration was not completed as data was collected as a quality assurance measure for the ESM-UBT kit.

## Background

Postpartum hemorrhage (PPH) is the leading cause of maternal mortality in low- and middle-income countries. Although reduction of maternal mortality has been a worldwide focus since 1990, a recent World Health Organization report on progress from 1990 to 2015 described that PPH-associated mortality remains unacceptably high, with 54,000 deaths in sub-Saharan Africa in 2015 alone [1, 2]. The majority of these deaths could have been averted with timely access to quality emergency obstetric care [2, 3].

First-line treatment for PPH includes administration of uterotonic agents. When hemorrhage persists, if available, alternative methods may be employed, including uterine balloon tamponade (UBT), non-pneumatic anti-shock trousers, and surgical interventions such as uterine artery embolization, B-Lynch compression sutures, and ultimately hysterectomy. In low-resource settings, access to surgical services is limited or non-existent, thus women with uncontrolled hemorrhage often die. Until recently, UBT devices were unavailable in these regions [2–4].

**Fig. 1:**
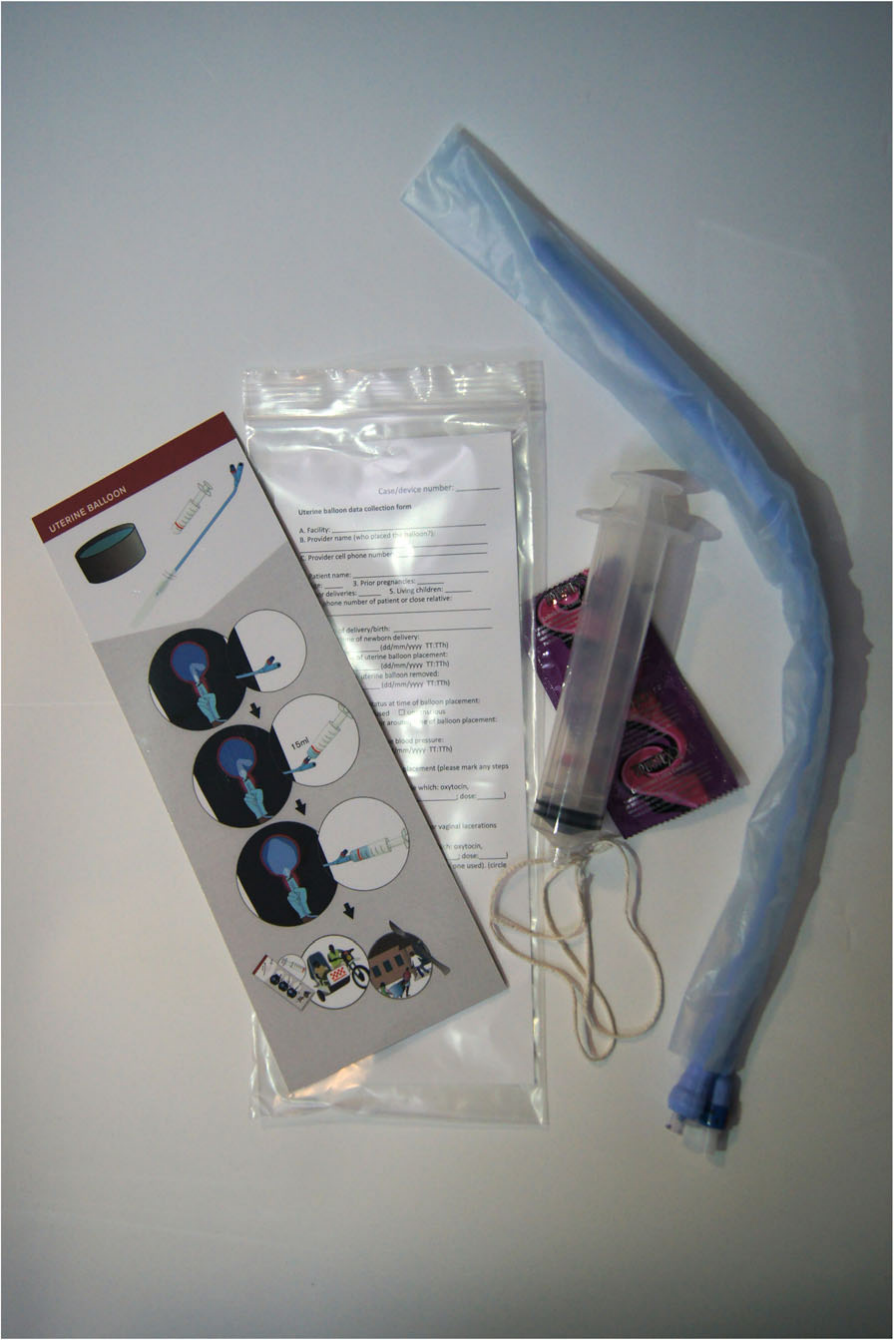
The ESM-UBT kit. Kit contents include: illustrated checklist, data collection card, cotton string, luer-lock syringe, condoms, and a size 24 urinary catheter. Source: Division of Global Health and Human Rights, Department of Emergency Medicine, Massachusetts General Hospital, Boston, MA, USA

Over the past 7 years our team has designed, developed, implemented, and refined a PPH evidence-based package utilizing an ultra-low-cost condom-based uterine balloon called Every Second Matters for Mothers and Babies–UBT (ESM-UBT) [5–8]. When compared with commercially available devices, ESM-UBT utilizes readily available low cost materials (Fig. 1). Data have been collected demonstrating efficacy of the package in arresting PPH and averting emergency hysterectomy [5–8]. The ESM-UBT package has been successfully implemented across all levels of the health systems in South Sudan, Kenya, Tanzania, Sierra Leone, Senegal, Zambia, Ghana and Nepal.

While the data to date support a high degree of efficacy of the ESM-UBT package in arresting uncontrolled PPH, with 98% survival overall, a critical and incomplete part of the equation defining overall impact is understanding the safety profile of the ESM-UBT device [6]. To date there have been limited follow-up data on the safety of UBT devices. A study from India followed 18 patients for 6 weeks after condom catheter tamponade placement and reported no complications [9]. Another report described 31 women who were followed for 4 months and longer after Rusch UBTs were placed, and also found no UBT-associated complications [10].

In this study, our goal was to better understand the safety profile of the ESM-UBT device by following all women who presented to our Kenya and Sierra Leone ESM-UBT facilities in whom an ESM-UBT device was placed for uncontrolled PPH.

## Methods

All women were enrolled who had an ESM-UBT device placed between September 2012 and December 2015, among the 92 facilities active with the ESM-UBT package in Kenya and Sierra Leone. Facilities were defined as active when 85% or more of their skilled birth attendants trained in ESM-UBT, ESM-UBT wall charts were mounted in each delivery area, and ESM-UBT manuals and devices were readily available. Data on improvised UBT kits placed by providers not trained on the ESM-UBT protocol were not included in this analysis (compared to our previous analysis on UBT efficacy) [6]. The UBT device was used within the context of the established national protocol for management of PPH when all other methods (including active management of the third stage of labor, emptying of the bladder, breastfeeding, administration of uterotonics, and manual removal of the placenta and blood clots) failed [6]. The ESM-UBT clinical pathway recommends a single dose of antibiotics, of the provider’s choice, to be administered after placement of an ESM-UBT device.

Data on adverse events/complications were collected via a multi-pronged approach to include telephone and in-person interviews with patients, village chiefs and community health workers; review of health facility records; and examination of data cards included in each of the ESM-UBT device kits. After a provider placed a UBT, the UBT provider filled out a data card and contacted the study coordinator. The study coordinators then visited the UBT facility to verify the data, formally interviewed the UBT provider and compared the data card with the patient’s chart. The data collection cards documented the kit tracking number, facility name, delivery date and time, interventions performed prior to UBT placement, mental status and vital signs at the time of UBT placement, effectiveness of hemorrhage control, steps taken after UBT placement, need for maternal resuscitation or transfusion, and adverse events. Adverse events/complications specifically sought included death, hysterectomy, uterine rupture, perineal or cervical injury, serious or minor infection, and latex allergy/anaphylaxis. Data were collected prospectively for the time interval between delivery and facility discharge throughout the entire study time period. Adverse events/complications data collection was extended to include follow-up at two and 6 weeks following facility discharge, beginning in October 2014. The field data collection team conducted formal follow-up interviews of UBT providers and mothers who had undergone UBT placement. Six-week or longer follow-up data were collected retrospectively for women who had an ESM-UBT device placed prior to October 2014. Three expert maternal health investigator physicians (AR, ME, and TB) analyzed each complication/adverse event and deliberated on whether there was a potential causal relationship associated with use of the ESM-UBT device. The three experts were instructed to err on the side of attributing possible causality if there was doubt in certainty. In cases of disagreement the three were asked to discuss and arrive at consensus on categorization.

Descriptive data analysis was performed utilizing Microsoft Excel 2015 (Seattle, WA, USA) and STATA 13.1 (College Station, TX, USA). This study was approved by the institutional review board of Partners HealthCare (Massachusetts General Hospital, Boston, MA, USA), the ethical review committee of Maseno University School of Medicine (Maseno, Kenya) and the Office of the Sierra Leone Ethics and Scientific Review Committee (Ministry of Health and Sanitation of Sierra Leone). Verbal informed consent was obtained in each case.

## Results

Among the 92 ESM-UBT active facilities, 201 women were treated with an ESM-UBT device for uncontrolled PPH in Kenya (154, 76.6%) and Sierra Leone (47, 23.4%) between September 2012 and December 2015. One-hundred and eighty-nine (94.0%) of the 201 women survived. All 201 women had their ESM-UBT devices placed at a health facility and 146 (76.0%) of 192 by a midlevel provider (clinical officer, midwife, nurse, or aide). One hundred and fifty-six (77.6%) of the 201 received a single dose of prophylactic antibiotics. The mean ages were 27.1 years in Kenya and 25.8 years in Sierra Leone. The median number of prior pregnancies was 2.0 in both countries. Thirty-nine (25.0%) of the hemorrhaging women in Kenya and 22 (46.8%) in Sierra Leone met criteria for advanced shock. Six (3.0%) of the 201 deliveries were via cesarean section (Table 1).

**Table 1:** Demographics of women receiving ESM-UBT device

	Kenya	Sierra Leone	Total
Patients (%)	154 (76.6)	47 (23.4)	201
Age, mean (range)	27.1 (16–44)	25.8 (16–41)	26.8 (16–44)
Prior pregnancies, median (range)	2 (0–13)	2 (0–7)	2 (0–13)
Prior deliveries, median (range)	2 (0–13)	2 (0–7)	2 (0–13)
Living children, median (range)	2 (0–13)	2 (0–5)	2 (0–13)
Location of delivery, n (%):			
Home	17/146 (11.6)	0 (0.0)	17/193 (8.8)
Facility	129/146 (88.4)	47 (100.0)	176/193 (91.2)
Level of provider placing UBT, n (%)^a^:			
Total	145/192 (75.5)	47/192 (24.5)	192
Doctor	43 (29.7)	3 (6.4)	46 (24.0)
Clinical officer/ clinical health officer	16 (11.0)	4 (8.5)	20 (10.4)
Midwife	86 (59.3)	12 (25.5)	98 (51.0)
State-controlled community health nurse	N/A	10 (21.3)	10 (5.2)
Maternal child health aide	N/A	18 (38.3)	18 (9.4)
Mode of delivery, n (%):			
Vaginal delivery	148/154 (96.1)	47 (100.0)	195/201 (97.0)
Cesarean section	6/154 (3.9)	0 (0.0)	6/201 (3.0)

^a^ Kenya designations: doctor, clinical officer, nurse. Sierra Leone designations: doctor, clinical health officer, midwife, state controlled community health nurse, maternal child health aide

### Adverse events/complications between delivery and facility discharge

All 12 maternal deaths in which ESM-UBT devices were placed occurred prior to facility discharge, and each case underwent detailed review by the respective ministries of health (county level in Kenya and national in Sierra Leone), by each of the facilities’ internal processes and by our own research team (Table 2). Nine (75.0%) of the 12 women who died were unconscious due to advanced hemorrhagic shock at the time the ESM-UBT device was placed and were not able to be resuscitated. One (8.3%) of the 12 maternal deaths was septic at the time of delivery with a fever of 40 degrees Celsius. She did not receive prophylactic nor treatment antibiotics, was found to have disseminated intravascular coagulopathy immediately after delivery, and died within an hour subsequent to delivery. Another woman (8.3%) had been hospitalized and treated for severe malaria over the week prior to the onset of her labor. Although the ESM-UBT device arrested a brief high-volume PPH and she received a single dose of prophylactic antibiotics, she was found dead during the early morning, 26 h after cessation of her hemorrhage. One (8.3%) of the maternal deaths was described as precipitous and the reported cause of death was “pulmonary embolism.”

**Table 2:** Causes of death among women in whom ESM-UBT devices were placed

	Kenya, n (%)	Sierra Leone, n (%)	Total, n (%)
Maternal deaths	6 (3.9)	6 (12.8)	12 (100.0)
Hemorrhagic shock	4 (2.6)	5 (10.6)	9 (75.0)
Sepsis	1(0.6)^a^	1(2.1)^a^	2 (16.7)
Possible pulmonary embolism	1 (0.6)	0 (0.0)	1 (8.3)

^a^ Sepsis symptoms were evident prior to delivery in both cases

One hundred and eighty-four (97.5%) of the 189 women who survived uncontrolled PPH had data recorded on adverse events/complications for the time interval between delivery and facility discharge (Table 3). Three (1.6%) of the 184 had a perineal or cervical injury: specifically, one (0.5%) had a “vulvo-vaginal laceration,” one (0.5%) had a “vaginal hematoma,” and one (0.5%) a “cervical laceration.” All three injuries were successfully repaired, and all three injuries were considered by the skilled birth attendants who performed the deliveries to have been caused by birth trauma and not the ESM-UBT device.

**Table 3:** Adverse events among PPH survivors, between delivery and discharge

	Kenya, n (%)	Sierra Leone, n (%)	Total, n (%)
Total cases	148 (78.3)	41 (21.7)	189 (100)
Data not available	5/148 (3.4)	0/41 (0.0)	5/189 (2.6)
Hysterectomy	1/143 (0.7)	0/41 (0.0)	1/184 (0.5)
Uterine rupture	0/143 (0.0)	0/41 (0.0)	0/184 (0.0)
Perineal or cervical injury	3/143 (2.1)	0/41 (0.0)	3/184 (1.6)
Serious infection	0/143 (0.0)	0/41 (0.0)	0/184 (0.0)
Minor infection	0/143 (0.0)	0/41 (0.0)	0/184 (0.0)
Latex allergy/anaphylaxis	0/143 (0.0)	0/41 (0.0)	0/184 (0.0)

One (0.5%) of the 184 underwent hysterectomy (unclear reasoning since hemorrhage reportedly had been arrested) and none experienced uterine rupture, serious or minor infection, or latex allergy/anaphylaxis in the time interval between ESM-UBT device placement and facility discharge.

Expert review of the adverse events and complications in the interval between delivery and facility discharge agreed that although unlikely, one of the twelve deaths (pulmonary embolism) and the three perineal injuries could have been related to use of the ESM-UBT device. No other adverse events potentially caused by an ESM-UBT device were thought to have occurred during this time interval.

### Adverse events/complications between facility discharge and 6 weeks post-delivery

One hundred and fifty-six (82.5%) of the 189 women who survived uncontrolled PPH subsequent to ESM-UBT device placement were followed for at least 6 weeks post-discharge (Table 4). Thirty-two (16.9%) women were lost to follow-up and one (0.5%) declined to participate. Three (1.9%) of the 156 experienced an adverse event/complication during the time interval between facility discharge and 6 weeks post-delivery. Two (1.3%) of the 156 reported minor infections over these 6 weeks. Both women had received a single dose of prophylactic antibiotics. One of these women labored for 15.5 h prior to cesarean section, after which an ESM-UBT device was placed for uncontrolled hemorrhage. Two weeks after delivery, she was diagnosed with and successfully treated for “mild endometritis.” The second woman was successfully treated with antibiotics for an “episiotomy wound infection,” diagnosed 2 weeks after delivery. Both women were successfully treated with antibiotics as outpatients and neither had signs of sepsis. One woman (0.6%) underwent hysterectomy 1 month after ESM-UBT placement due to “continued bleeding.”

**Table 4:** Adverse events among PPH survivors between discharge and six-week follow-up

	Kenya, n (%)	Sierra Leone, n (%)	Total, n (%)
Total cases	148 (78.3)	41 (21.7)	189 (100)
Lost to follow-up	32 (21.6)	0 (0.0)	32/189 (16.9)
Declined to participate	1 (0.7)	0 (0.0)	1/157 (0.6)
Minor infection	2 (1.7)	0 (0.0)	2/156 (1.3)
Hysterectomy	1 (0.9)	0 (0.0)	1/156 (0.6)
Total adverse events	3 (2.6)	0 (0.0)	3/156 (1.9)

Review of the adverse events and complications in the interval between facility discharge and 6 weeks post-delivery agreed that the one case of endometritis could have been associated with use of the ESM-UBT device. No other adverse events potentially caused by an ESM-UBT device were reported during this time interval.

## Discussion

While efficacy of the ESM-UBT package has previously been reported, this investigation aimed to examine the safety of the ESM-UBT device [5–8]. Of the 201 women treated with an ESM-UBT device in this study, 189 (94.0%) survived, of which in-facility safety data were available for 184 (97.4%) and six-week follow-up in 156 (82.5%). Immediate postpartum, in-facility adverse outcomes and complications included 12 (6.0%) deaths, three (1.6%) of 184 that experienced perineal or cervical injuries and one (0.5%) of 184 that underwent hysterectomy. In the 156 women for whom follow-up data between facility discharge and 6 weeks post-delivery were available, two (1.3%) experienced minor infections and one (0.6%) underwent hysterectomy.

Nine (75.0%) of the 12 women who died were in advanced hemorrhagic shock at the time of ESM-UBT device placement and two were septic (one from malaria) prior to delivery (Table 2). The ESM-UBT device appeared to play no role in the deaths of these 11 women. Although seeming unlikely, the three experts thought that it was not possible to exclude a potential association between the one precipitous death that was assigned pulmonary embolism (no autopsy performed) and the use of the ESM-UBT device.

Hemorrhagic shock remains the number one killer of pregnant women in the world today, accounting for as many as 130,000 deaths annually [11]. Among the nine hemorrhagic shock deaths in this study, the consistent theme was very late attempts at intervention. Septic shock occurs in 0.002–0.01% of all deliveries worldwide and accounts for 12–13% of maternal deaths in the United States [12–14]. While an ESM-UBT device could conceivably contribute to infection and sepsis since it involves introduction of a foreign body into the uterus, neither of the maternal deaths from sepsis in this study appeared related to the ESM-UBT device, given that symptoms clearly preceded device placement. Overall, of the 156 women who were followed for at least 6 weeks, no cases of ESM-UBT-associated sepsis were identified.

In highly resourced settings, pulmonary embolism has an overall incidence in pregnancy of one per 7000 and has been described responsible for 9–10% of maternal deaths [12, 13]. However, in low resource poor settings, without autopsy and/or advanced imaging, diagnostic certainty regarding pulmonary embolism is low; thus causation from the ESM-UBT device cannot be entirely eliminated.

The baseline incidence of delivery-associated severe vaginal lacerations is 4–5%, perineal hematomas 0.1–0.3%, and cervical lacerations 0.2% [13, 15–17]. The finding of three (1.6%) perineal injuries among the 184 women who received ESM-UBT devices in our study was a lower rate than what would have been expected in the general population of women who deliver. Additionally, our skilled birth attendants and our three experts felt certain that the ESM-UBT device did not cause these three perineal injuries.

Two women (1.0%) in our study underwent non-emergency hysterectomy subsequent to ESM-UBT placement, however, neither appeared related to ESM-UBT device use. In fact, the hysterectomy rate in this population of 201 women with uncontrolled PPH was low compared to expected rates of 3.8–35.0% [9, 10, 18–21]. These findings further supported our previously reported findings on the ability of ESM-UBT to avert hysterectomy [8].

During the six-week follow-up period, two women (1.3%) were noted to have minor infections despite having been administered a single dose of prophylactic antibiotics. One was diagnosed with mild endometritis and one an infection of her episiotomy site. It was unlikely that the ESM-UBT device contributed to the episiotomy wound infection, but, quite possible, despite her prolonged labor and cesarean section, that use of the ESM-UBT device contributed to the one case of suspected endometritis. At baseline, endometritis is diagnosed after 1–3% of vaginal deliveries and is ten times more common following cesarean section [22]. In our series of 156 women followed out to 6 weeks, the one (0.6%) case of endometritis falls below the expected baseline among the general population of women who deliver.

This study is the largest case series to date examining the safety of a condom UBT device. A lack of absolute certainty of the diagnoses was one of the primary limitations. A second limitation was that the authors examined the data and attributed causation, thus inviting potential reporting bias. To mitigate this bias, the investigators were intentionally inclusive in assigning potential association and/or causation. Another limitation was the incomplete follow-up out to 6 weeks post-delivery despite the multi-pronged approach to data collection. Almost all women lost to follow-up were from the nomadic tribes along the border with Somalia, primarily in Garissa County, Kenya. However, given that the multi-pronged data collection included direct communication with, and interviews of, rural community health workers and local health providers in all the settings where ESM-UBT devices were placed, it was unlikely any deaths were missed. Another limitation was the potential for recall bias when women were asked to recount their experience months subsequent to placement of an ESM-UBT device. However, we expected that most women would recall such injuries, infections or other complications even after the passage of time. Additionally, although it is difficult to draw causal inferences about adverse events in a case series study design, the authors believe that a randomized controlled trial would have been unethical.

Although it is impossible to assign causation between ESM-UBT device placement and the adverse events with complete certainty, it appears that, at worst, the ESM-UBT device was associated with one death, three perineal injuries, and one case of mild endometritis among the women in this study. Since the adverse events and complication rates were not above expected baseline in women among the general population of deliveries, and since most of the potential associations between these adverse events and the ESM-UBT device were not likely, it appears that the risk of ESM-UBT device-associated harm is low when used in women with uncontrolled PPH. In this case series, the ESM-UBT device appears safe for use in women with uncontrolled PPH.

## Conclusion

In this case series, the ESM-UBT device appears safe for use in women with uncontrolled PPH. Future studies on UBT should seek to further define harm versus benefit and optimal use characteristics, clarify the role of antibiotics, develop models for sustainability and scale, and identify additional opportunities for ending maternal death and disability from PPH.

## Abbreviations

ESM-UBT: Every second matters for mothers and babies-uterine balloon tamponade
PPH: Postpartum hemorrhage
UBT: Uterine balloon tamponade
